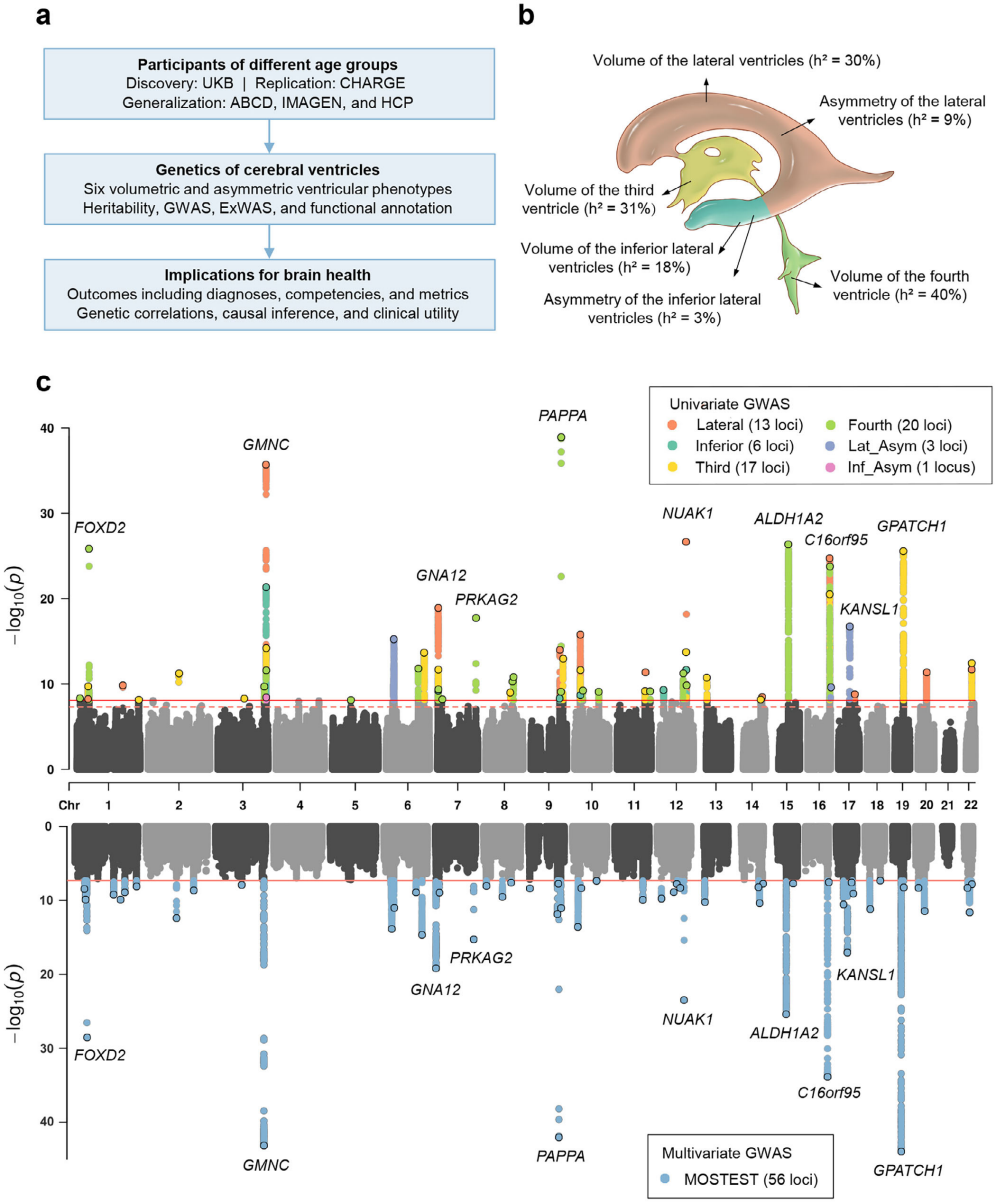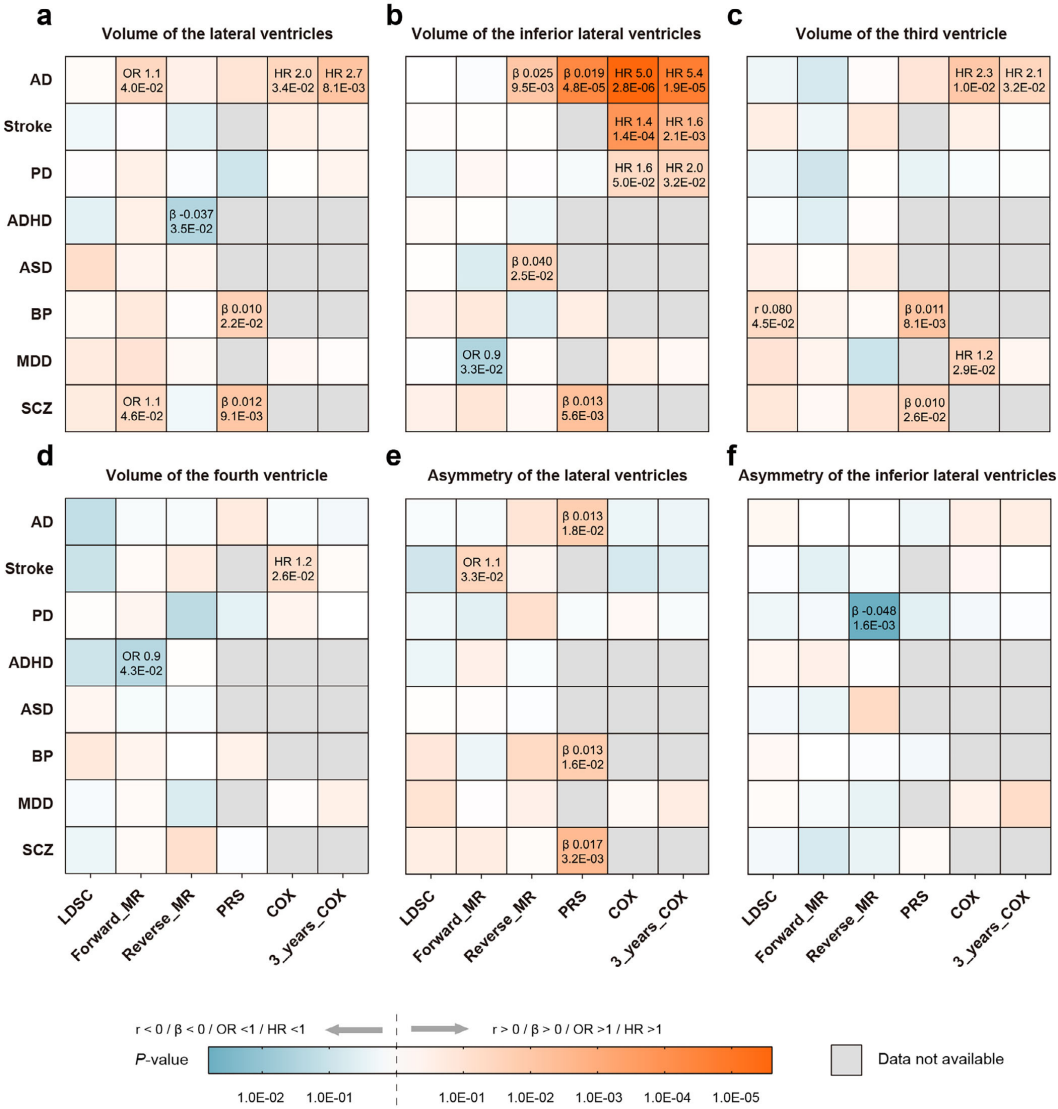# Genetic architectures of cerebral ventricles and their overlap with neuropsychiatric traits

**DOI:** 10.1002/alz70855_102636

**Published:** 2025-12-23

**Authors:** Yi‐Jun Ge, Bang‐Sheng Wu, Yi Zhang, Jin‐tai Yu

**Affiliations:** ^1^ Huashan hospital, Fudan University, Shanghai, China

## Abstract

**Background:**

Cerebral ventricles are recognized as windows into brain development and disease, yet their genetic architectures, underlying neural mechanisms, and utility in maintaining brain health remain elusive.

**Method:**

Here we aggregated genetic and neuroimaging data from 61,974 participants (age range, 9 to 98 years) in five cohorts to elucidate the genetic basis of ventricular morphology and examined their overlap with neuropsychiatric traits.

**Result:**

Genome‐wide association analysis in a discovery sample of 31,880 individuals identified 62 unique loci and 785 candidate genes associated with ventricular morphology. We replicated over 80% of loci in a well‐matched cohort of lateral ventricular volume. Gene set analysis revealed enrichment of ventricular‐trait‐associated genes in biological processes and disease pathogenesis during both early brain development and degeneration. We explored the age‐dependent genetic associations in cohorts of different age groups to investigate the possible roles of ventricular‐trait‐associated loci in neurodevelopmental and neurodegenerative processes. We describe the genetic overlap between ventricular and neuropsychiatric traits through comprehensive integrative approaches under correlative and causal assumptions. We propose the volume of inferior lateral ventricles as a heritable endophenotype to predict the risk of Alzheimer's disease (AD), which might be a consequence of prodromal AD.

**Conclusion:**

Our study provides an advance in understanding the genetics of cerebral ventricles and demonstrates the potential utility of ventricular measurements in tracking brain disorders and maintaining brain health across the lifespan.